# Benefits and limitations of different study designs for long‐acting antiretroviral therapy among people living with HIV with viremia

**DOI:** 10.1002/jia2.26093

**Published:** 2023-07-13

**Authors:** Monica Gandhi, Jean Nachega, Veronica Miller, Timothy Wilkin

**Affiliations:** ^1^ Division of HIV, Infectious Diseases and Global Medicine Department of Medicine, University of California San Francisco (UCSF) San Francisco California USA; ^2^ Departments of Epidemiology, Infectious Diseases and Microbiology University of Pittsburgh School of Medicine Pittsburgh Pennsylvania USA; ^3^ Departments of Epidemiology and International Health Johns Hopkins Bloomberg School of Public Health Baltimore Maryland USA; ^4^ Division of Infectious Diseases Department of Medicine, Faculty of Medicine and Health Sciences Stellenbosch University Cape Town South Africa; ^5^ Forum for Collaborative Research Washington DC USA; ^6^ Weill Cornell Medicine ‐ Division of Infectious Diseases New York USA

1

Although there are highly effective options for once‐daily oral antiretroviral therapy (ART) to treat HIV, not all patients living with HIV on ART are virologically suppressed. Adherence barriers to oral regimens are common and span individual and structural factors, including stigma, forgetting, unstable housing, food insecurity, insurance lapses or prohibitive co‐pays, mental illness, substance use, transportation challenges and stock‐outs. Long‐acting (LA) injectable agents administered monthly or less frequently could circumvent some of these barriers.

LA agents have been developed for contraception and other disease states like psychiatric illness. For HIV infection, LA treatment is relatively new. The U.S. Food and Drug Administration (FDA) approved a combination of two injectable antiretroviral medications, cabotegravir (CAB) and rilpivirine (RPV), for administration every 4 weeks in both ART‐naïve and ART‐experienced patients in January 2021; a higher dose of each given every 8 weeks was approved in March 2022. The approvals were based on registrational trials of LA CAB/RPV in treatment‐naïve and experienced participants who were first virologically suppressed on oral regimens [1, 2, 3]. In an updated analysis of the registrational trials out to 152 weeks, the confirmed virologic failure rate was low (1.4%) [4]. A recently presented study confirmed low rates of virologic failure (0.4%) and high treatment satisfaction at 12 months with a switch to LA CAB/RPV from bictegravir/tenofovir alafenamide/emtricitabine (BIC/TAF/FTC) [5]. Two demonstration projects in Europe found high rates of maintenance of virologic suppression and treatment satisfaction when switched to injectable agents [6, 7].

All of these studies started LA CAB/RPV in virologically suppressed patients. However, given the difficulties with adherence in multiple contexts worldwide, studies using LA‐ART in adherence‐challenged patients are needed. A demonstration project conducted at the Ward 86 HIV Clinic at San Francisco General Hospital, which serves a publicly insured or uninsured population of people with HIV (PWH) in San Francisco, provides the most extensive experience to date: 133 patients received LA CAB/RPV; 76 (57%) of whom were virologic suppressed on oral ART and 57 (43%) with viremia off of ART due to adherence challenges [8]. The cohort was diverse: 68% non‐White; 88 (66%) unstably housed; and 44 (33%) endorsed substance use. All patients started on injectable ART who had achieved virologic suppression on an oral regimen remained suppressed. All but two of the viraemic patients were suppressed on LA CAB/RPV over a median of 26 weeks (2–42), providing an overall virologic suppression rate in the cohort of 98.5%, similar to that in registrational clinical trials. The rate of suppression among initially viraemic patients was 96.5%. Of note, the Ward 86 demonstration project included frequent outreach and treatment support services. While these results are not directly comparable to data from randomized clinical trials, they are encouraging in demonstrating that administration of LA CAB/RPV *can* lead to virologic suppression in unsuppressed patients.

Discussions around the use of LA CAB/RPV before and after its approval have centred around the idea of equity and extending the use of this combination treatment to adherence‐challenged and hard‐to‐reach populations, including those with marginal housing, individuals experiencing incarceration and adolescents [9, 10, 11]. However, a randomized controlled trial (RCT) comparing LA CAB/RPV in patients with viremia on oral ART has never been performed. The AIDS Clinical Trials Group (ACTG) is studying LA CAB/RPV among adherence‐challenged patients who have demonstrated difficulty achieving virologic suppression on oral ART. In this RCT (A5359), participants are being randomized to a standard‐of‐care oral ART arm versus an arm using LA CAB/RPV after the achievement of virologic suppression on oral ART with adherence interventions and incentives. Although this study will reveal important insights into treating adherence‐challenged populations who are able to first suppress on oral therapy (with incentives), the utility of treating viraemic patients with LA CAB/RPV cannot be defined by the A5359 study design.

The U.S. FDA usually approves medications based on evidence from randomized trials. However, patients with ongoing viremia are often not able to suppress on an oral ART regimen, which can make an RCT difficult to enrol and a likely set‐up for failure in the oral ART arm. A recent modelling study showed that only 22% of patients with long‐standing adherence challenges and viremia are likely to suppress on oral ART, even with wrap‐around services [12]. Therefore, examining the question of using LA CAB/RPV via an RCT design does not meet the criteria for equipoise, is not likely to be feasible and may even be unethical for those randomized to oral ART.

In the case of a “rare condition,” the U.S. FDA allows single‐arm trials to determine the efficacy of a medication in the population [13]. For instance, the prevalence of multidrug‐resistant (MDR) HIV‐1 in the United States is low and the disease state is, therefore, rare. The entry inhibitor, ibaluzimab, was approved by the FDA for use in MDR HIV based on the results of a single‐group, open‐label study with a total of 31 participants enrolled [14]. Although adherence difficulties are common, the population who cannot take oral ART and will only agree to be on ART if prescribed as monthly or bimonthly injectables is relatively rare. Therefore, we would argue that a single‐arm trial to show the effectiveness of LA CAB/RPV among viraemic patients who will not take oral ART is not only justifiable, but the most feasible and ethical design.

Although important for market introduction, the drug label is not the only source of evidence for clinical practice and reimbursement. Treatment guidelines from reputable sources can influence which medications are reimbursed. In HIV, the Department of Health and Human Services Panel on Antiretroviral Treatment Guidelines for Adults and Adolescents in the United States [15] serves this purpose. In contrast to the stringent requirements for regulatory approval and indication as stated on the drug label, treatment guideline panels review and grade all available evidence. A larger, multi‐centre, single‐arm trial for LA CAB/RPV in viraemic patients than what has been presented to date from Ward 86 could lead to this addition to the guidelines, which will help prescribing providers garner insurance approval in the United States. The trial design could involve examining feasibility and acceptability, in addition to effectiveness and safety, which would aid in implementation in real‐world settings.

We thereby recommend the consideration of a well‐conducted, multi‐centre, single‐arm trial examining LA CAB/RPV in viraemic participants. The details of the study design should involve intensive consultation with the community as well as clinicians with expertise in serving these communities to examine safety, efficacy, acceptability and feasibility.

Whether or not LA‐ART will change population health outcomes depends on the willingness of the HIV community to test this strategy in viraemic patients in order to generate data for treatment guideline panels and regulatory authorities. To truly reach the U.S. End the HIV Epidemic (EHE) goals, we need a way to harness innovative strategies to help patients with viremia achieve virologic suppression. LA ART can be the “game changer” to help achieve EHE goals, and innovative study designs to rapidly evaluate this important new strategy among viraemic patients are indicated.

## COMPETING INTERESTS

None of the authors reports any relevant competing interests.

## AUTHORS’ CONTRIBUTIONS

MG drafted the original manuscript; JN, VM and TW edited the manuscript, added references and reviewed the final version.
